# Sports press: an explanatory and identity scheme

**DOI:** 10.3389/fsoc.2024.1390944

**Published:** 2024-05-09

**Authors:** Bechir Nasri, Ines Souid

**Affiliations:** ^1^Higher Institute of Sport and Physical Education Kef, University of Jendouba, Tunis, Tunisia; ^2^University of Lyon, Lyon 1 L-VIS EA 74 28 SFR CRIS FED 4272, Lyon, France

**Keywords:** sports press, identity, diagram, analysis, exploration

## 1 Introduction

The media discourse on sports often revolves around athletes, who are central elements of journalistic texts. This narrative is typically based on various elements, including actors, roles and functions, and typical interactions between these actors. Athletes play a central role in media coverage of sports by highlighting their performances, personal stories, and opinions. This visibility is ensured by the coverage of major sporting events, interviews in specialist magazines, television reports, and coverage of transfers and contracts, as well as their presence on social media.

Various journalistic genres, such as match reports, athlete profiles, analyses, interviews, and editorials, respond to different proximal mechanisms. They enrich the reading experience by offering a variety of information, analysis, and perspectives, allowing readers to further engage with sports content.

Individualization in media coverage of athletes often manifests through the presentation of their personalities, achievements, and lives outside of sports, similar to that of politicians in political marketing. For example, figures such as Michael Jordan, Usain Bolt, and Serena Williams have been publicized not only for their exceptional sporting performances but also for their charisma, lifestyle, and partnerships with renowned brands. This spotlight creates a star image, establishing emotional connections with the public and reinforcing their status as global icons.

On the other hand, Patrick Mignon specifically reviews the sporting and public performances of athletes. Figures of projection undergo various logics of reification and serve as emblems for territorial arguments, national selections, and local teams, as discussed by Bonnet (2007). The reification of athletes for sporting, territorial, or editorial purposes transforms them into simple tools or symbols, dehumanizing and reducing them to instruments serving external objectives. This leads to increased pressure and a loss of authenticity, which can affect their self-esteem. It is crucial to recognize their humanity and individuality to preserve their wellbeing and identity beyond sport, as mentioned by Attali and Lemonnier (2011).

Additionally, the relationship between the discourse producer, such as sports media, and the audience is a complex interaction that shapes media representations of athletes. The media tends to reify athletes by presenting them according to cultural archetypes and pre-existing norms. This reification can simplify athletes' identities and perpetuate social stereotypes. As a result, audiences may perceive athletes through these media lenses, thereby influencing their understanding and appreciation of the sport and its stakeholders.

This article aims to explore the way in which athletes are represented in the media, highlighting their central role in journalistic storytelling. Our objective is to demonstrate how media discourse shapes and conveys explanatory and identity schemes around athletes, thus influencing public perception. By examining these dynamics, we contribute to a better understanding of media discourse on sports by highlighting the complex links between the media, athletes, and the public.

## 2 Identity assignments and social issues

Due to the very polysemous nature of the term identity, whose definitions oscillate between more or less strong or weak conceptions (Bessone et al., 2015), talking about identification logic makes it possible to better specify the purpose of this delivery, which consists of analyzing the forms of categorical identity assignment present in the sports press and the sports sections of the general press without reifying them. For sociologists, identity is understood as a successful fiction resulting from a social process of construction and imposition [Tap, (n.d.)]. As Brubaker (2001, p. 70) suggests, the process of establishing identity through discourse is essential in sports media because it helps to crystallize and normalize certain social identities. This is done by stereotyping and essentializing athletes based on their gender, race, and class, prioritizing these identities, and naturalizing them.

First, these speeches and representations are part of the journalistic field (Champagne, 2006), where sports journalists and those responsible for sports sections occupy a dominant position. Their social origin is less favored than their colleagues, less endowed with cultural capital (Diana, 2013), and they experience forms of professional socialization that do not promote a critical distance with regard to their sources (Fraysse and Mennesson, 2016, 2017). In the case of the written sports press, which is in a subordinate position compared to television sports broadcasts, working conditions influence the ability to question stereotypes, which are integral to the sense of belonging to the environment (Marchetti, 2002).

Thus, as Montañola (2011) shows, the presentation of sportsmen and sportswomen can sometimes move away from the expected stereotypes. These variations in the diffusion of hegemonic models have been identified by Connell and Messerschmidt (2005), who highlight local forms of masculinity or femininity. This process is observed in the case of journalists from the VTT press: those who are most endowed with cultural capital (Fraysse and Mennesson, 2016, 2017).

Financial issues and the overlapping of the journalistic and economic fields have a significant impact on media representations of athletes. For example, advertising contracts and sponsorships often influence media coverage, favoring some athletes over others. Additionally, the most profitable sports typically receive disproportionate attention, which can reinforce inequities in athlete representation. By understanding these dynamics, readers can challenge media narratives and promote more equitable and inclusive coverage of sports (Dargelos and Marchetti, 2000; Rowe, 2006).

The body plays a central role in the socialization of the biological and the biology of the social (Bourdieu, 1998), forming the basis of hierarchies between sexes and racial categories (Guillaumin, 1992). The sporting world can thus be considered a stronghold of virility (Elias and Dunning, 1986), just as it constitutes a place of expression for social and racial relations (McKay and Laberge, 2006).

Largely imbued with the culture of the field, sports journalists often naturally adopt these forms of identity assignment (Schoch and Ohl, 2011; Fraysse and Mennesson, 2016, 2017). For athletes, markers of gender, hyperfemininity, and eroticization often take precedence over the presentation of their performances (Knoppers and Elling, 2001; Buysse and Embser-Herbert, 2004; Hardin and Shain, 2005; Schoch, 2008; Fraysse and Mennesson, 2009). On the men's side, masculinity and the staging of violence constitute the modes of presentation commonly used for athletes (Messner et al., 2000; Sabo, 2005). Thus, the sporting successes of black athletes are presented more as the consequence of natural physical qualities (Rowe et al., 2000; Knoppers and Elling, 2001; Mennesson and Sorignet, 2007). In sports journalism, attributing categorical identity is common to contextualize athletes' performances. For instance, labeling Serena Williams as a “tennis champion” ties her identity to her gender. Similarly, mentioning Usain Bolt's Jamaican nationality reinforces his racial identity, while highlighting Cristiano Ronaldo's luxury real estate investments associates his identity with his social class. While this can aid in contextualizing stories, it is crucial to recognize that these attributions can also perpetuate stereotypes and biases. Without analyzing here the scope and diversity of the modes of reception of media discourses (Hall, 1973; Bouillin-Dartevelle, 1993), a subject that would require a specific journal delivery, we nevertheless consider that the latter can contribute to the logic of identification, allowing readers to position themselves in the social world. In other words, the media participate in what Lahire (2001) calls socialization by ideological inculcation.

## 3 The role of the contract

The reading contract emerges in response to the positioning of the press vis-à-vis its competitors and its public and is based on the materiality of the media system (Dayan, 2012). At the heart of this relationship between the press and its readership is the materiality of the media system, which defines the terms of this tacit agreement (Kennedy and Zamuner, 2006). Visual elements such as emblematic photographs of motorsport culture (Jeanneret and Patrin-Leclère, 2004) or notable moments such as the golden bowl, analyzed by Mélie Fraysse, only take on their meaning in the context of a specific social logic. Thus, clarifying the notion of the reading contract and understanding its implications in the relationship between the press and its readership are essential to understanding how this contract influences the content and reception of sports media coverage.

These logics of proximity implemented in the specialized press establish a bond of trust (Verón, 1985), allowing the model readership to meet the empirical readership, a point that Mélie Fraysse developed in her analysis of the continuum existing between the editors and the readers of the mountain bike reviews: just like proximity, the terrain constitutes a preconstructed foundation of journalistic discourse (Ringoot and Rochard, 2005).

The sociological characteristics of journalists, such as their social origin and cultural capital, shape the editorial choices of sports media, influencing the topics covered and the perspectives presented. This creates diversity in the representation of athletes, reflecting the different visions of journalists and impacting the public's perception of athletes (Ringoot and Rochard, 2005).

Elise Pons thus shows that the titles of the articles are hardly accessible for those who do not know the equestrian world, and Mélie Fraysse shows that the gender models are not unanimously shared. Reception can be conceived as a production of meaning (Verón, 2001) based on indices of meaning that offer a certain type of reading, a game of appropriation constructed, among other things, by the relationship established with the readership, and therefore, a certain type of communication. These identity links between the medium and its audience are subject to feedback logic; this constructs a reading community thanks to specific enunciative postures, these enunciative postures finding their source in a certain number of the constituent traits of these communities. A well-known phenomenon among rhetoricians is that “postulates concerning the other influence the form of the text being produced or what is inferred from this form” (Hymes, 1982); in other words, language choices are made according to the context, which in turn is constructed by said language choices. This reading contract, closely linked to the competitive logic of the press, is therefore coupled with a communication contract (Charaudeau, 2017). In other words, the reading contract represents the mutual expectations between readers and the media for quality content, while the communication contract concerns the relationship between the media and advertisers to guarantee advertising visibility. These contracts are closely linked because the media must satisfy both their audiences and their advertisers to remain competitive.

## 4 Conclusion

Constructed as a character, the high-level sportswoman or sportsman, through their notoriety, “allows all forms of identification, especially in mass cultural productions” (Lits, 2009), variable identification giving rise to journalistic production outside of the specialized sector. This is where the discourse on sport and the discourse on sportsmen and women are separated: it is as individuals that the latter are potentially bearers of identity traits attributed, valued, and often unique, not only for the readability of the articles but also to respond to the supposed expectations and imaginations of readers. In summary, the representations of athletes in the press are at the center of a relationship between the speaker, the recipient, and the world described, a relationship which can be analyzed in terms of negotiation of cultures and reading links.

The strengths of the article lie in its in-depth analysis of the relationship between sports media and the construction of athletes' identities, highlighting the impact of journalists' sociological characteristics. However, weaknesses include a possible insufficient analysis of media bias and a lack of consideration of athletes' perspectives on their representation in the media.

## Author contributions

BN: Writing – original draft, Writing – review & editing. IS: Writing – review & editing.
